# Homologous recombination repair genetic testing variables and diagnostic paths for prostate cancer patients: a multicenter cohort study

**DOI:** 10.1093/oncolo/oyaf395

**Published:** 2025-12-02

**Authors:** Lorena Incorvaia, Mattia Puglisi, Marco Maruzzo, Giulia Mammone, Orazio Caffo, Giuseppe Procopio, Lorenzo Antonuzzo, Mimma Rizzo, Vincenza Conteduca, Carlo Messina, Sarah Scagliarini, Brigida Maiorano, Matteo Santoni, Gaetano Facchini, Helga Lipari, Luigi Formisano, Marco Stellato, Umberto Basso, Sabrina Rossetti, Eleonora Lai, Carlo Carmelo Arcara, Enrico Bronte, Tancredi Didier Bazan Russo, Giovanni Colletta, Valerio Gristina, Francesco Pepe, Umberto Malapelle, Daniele Santini, Ilaria Depetris, Massimo Di Maio, Giuseppe Badalamenti, Sergio Bracarda, Antonio Russo

**Affiliations:** Department of Precision Medicine in Medical, Surgical and Critical Care (Me.Pre.C.C.), Section of Medical Oncology, University of Palermo, Palermo, 90127, Italy; Department of Precision Medicine in Medical, Surgical and Critical Care (Me.Pre.C.C.), Section of Medical Oncology, University of Palermo, Palermo, 90127, Italy; Oncology 3 Unit, Istituto Oncologico Veneto—IOV IRCCS, Padova, 35128, Italy; Medical and Translation Oncology, Azienda Ospedaliera S. Maria, Terni, 05100, Italy; Department of Medical Oncology, Santa Chiara Hospital, Trento, 38122, Italy; Department of Medical Oncology, Fondazione IRCCS Istituto Nazionale dei Tumori di Milano, Milan, 20133, Italy; Clinical Oncology Unit, Careggi University Hospital, Florence, 50134, Italy; Division of Medical Oncology, A.O.U. Consorziale Policlinico di Bari, 70124, Italy; Department of Medical and Surgical Sciences, Unit of Medical Oncology and Biomolecular Therapy, University of Foggia, Foggia, 71121, Italy; Oncology Unit, ARNAS Civico, Palermo, 90127, Italy; Division of Oncology, Azienda Ospedaliera di Rilievo Nazionale A. Cardarelli, 80131, Italy; Department of Medical Oncology, IRCCS San Raffaele Hospital, Milan, 20132, Italy; Oncology Unit, Macerata Hospital, Macerata, 62100, Italy; Medical Oncology Unit, SM delle Grazie Hospital, Pozzuoli, 80078, Italy; Medical Oncology Unit, Ospedale Cannizzaro, Catania, 95126, Italy; Department of Clinical Medicine and Surgery, University of Naples “Federico II”, Naples, 80131, Italy; Department of Medical Oncology, Fondazione IRCCS Istituto Nazionale dei Tumori di Milano, Milan, 20133, Italy; Oncology 3 Unit, Istituto Oncologico Veneto—IOV IRCCS, Padova, 35128, Italy; Division of Medical Oncology, Department of Uro-Gynaecological Oncology, Istituto Nazionale Tumori ‘Fondazione G. Pascale’-IRCCS, Naples, 80131, Italy; Oncology 3 Unit, Istituto Oncologico Veneto—IOV IRCCS, Padova, 35128, Italy; Medical Oncology Unit, La Maddalena Hospital, Palermo, 90146, Italy; Medical Oncology Unit, Ospedale Cervello Villa Sofia, Palermo, 90146, Italy; Department of Precision Medicine in Medical, Surgical and Critical Care (Me.Pre.C.C.), Section of Medical Oncology, University of Palermo, Palermo, 90127, Italy; Department of Precision Medicine in Medical, Surgical and Critical Care (Me.Pre.C.C.), Section of Medical Oncology, University of Palermo, Palermo, 90127, Italy; Department of Precision Medicine in Medical, Surgical and Critical Care (Me.Pre.C.C.), Section of Medical Oncology, University of Palermo, Palermo, 90127, Italy; Department of Public Health, University Federico II of Naples, Naples, 80131, Italy; Department of Public Health, University Federico II of Naples, Naples, 80131, Italy; Division of Oncology, Department of Radiological, Oncological and Pathological Science, Policlinico Umberto I, Sapienza Università di Roma, 00161, Italy; Department of Oncology, University of Turin, AOU Città della Salute e della Scienza di Torino, 10126, Italy; Department of Oncology, University of Turin, AOU Città della Salute e della Scienza di Torino, 10126, Italy; Department of Precision Medicine in Medical, Surgical and Critical Care (Me.Pre.C.C.), Section of Medical Oncology, University of Palermo, Palermo, 90127, Italy; Medical and Translation Oncology, Azienda Ospedaliera S. Maria, Terni, 05100, Italy; Department of Precision Medicine in Medical, Surgical and Critical Care (Me.Pre.C.C.), Section of Medical Oncology, University of Palermo, Palermo, 90127, Italy

**Keywords:** *BRCA*, homologous recombination deficiency, homologous recombination repair, prostate cancer

## Abstract

**Background:**

Evidence on homologous recombination repair (HRR) mutation prevalence in prostate cancer (PC) patients and the diagnostic testing path to guide treatment remains limited outside of clinical trials. The objective of this study was to investigate the DNA source, type of tumor tissue, timing for testing in the patient’s disease course, and rate of conclusive results in a real-world population.

**Patients and Methods:**

This was an observational, cohort study, involving 20 Italian cancer centers. The study population included consecutive PC patients undergoing germline, tumor, and/or plasma circulating tumor DNA (ctDNA) to profile HRR genes between January 1, 2020 and January 31, 2025.

**Results:**

Among 1400 PC patients included, 248 (17.7%) showed (likely)pathogenic variants (PVs) in the HRR genes. Most HRR testing was conducted during the metastatic castration-resistant PC (mCRPC)(779, 62.8%). The rate of conclusive results was 89.7% and varied widely according to the type of tumor tissue. The prevalence of HRR alterations was 18.1% in the mCRPC and 13.8% in the hormone-sensitive PC (*P* = .06). The concordance between tumor testing and ctDNA was 83.9%. Interestingly, 4.6% reported ctDNA testing positive but tumor testing negative, leading to important therapeutic implications. The prevalence of positive ctDNA testing was 47.4% vs 10.5%, for testing within 1 month or over 3 months, respectively, from the initiation of a new line of therapy.

**Conclusion:**

This large real-world study, through the workflow adopted by clinicians for HRR genomic testing, provides novel insights into the variables influencing the success rate of genomic testing.

Implications for Practice In this report, we looked at *BRCA1/2* and other HRR gene mutations in a large real-world population of PC patients. We found that several variables associated with type and time of genomic testing may affect the results. Optimizing *BRCA*/HRR testing, choosing the optimal DNA source, disease state, and timing for tissue and blood collection, can allow to draw of virtuous workflows for molecular testing approaches to maximize the detection and understanding of clinically relevant HRR gene alterations in PC patients, thus impacting disease outcomes and informing future clinical practice.

## Introduction

Understanding the molecular landscape of prostate cancer (PC) has become increasingly complex in recent years. Genetic and genomic testing to identify germline and tumor alterations in Breast Cancer Susceptibility Gene 1 (*BRCA1*) and Breast Cancer Susceptibility Gene 2 (*BRCA2*) has rapidly emerged as a critical tool to inform clinical decision-making in metastatic PC (mPC).^1^^,^^2^  *BRCA1/2* (likely)pathogenic variant (PV) have been consistently associated with aggressive phenotypes and adverse clinical outcomes,^3–6^ providing prognostic information and possible practice changes in the care paths.^7–11^

The prevalence of homologous recombination repair (HRR) PV type varies across the PC spectrum.^12^ Differently from HRR-associated breast and ovarian cancers, in PC patients somatic HRR gene alterations are more frequent than germline, with a prevalence in mPC significantly higher (approximately 12%) than localized disease (approximately 5%), and HRR gene alterations in up to 15%-25% of metastatic castration-resistant PC (mCRPC) patients.^13–15^

This relatively high prevalence of HRR gene alterations in mPC patients has been usefully exploited using synthetic lethality between *BRCA1/2* PVs and the poly (ADP) ribose polymerase (PARP)-inhibitors (PARPis).^16–20^ With the introduction of PARPis, and the novel combination of PARPi plus an androgen receptor pathway inhibitor (ARPI),^21–23^ several oncological and urological Societies now move to testing all mPC patients for somatic and germline HRR PVs in the therapeutic setting.^24^ However, published data on *BRCA1/2,* and other HRR gene alteration prevalence, are very heterogeneous, derive primarily from clinical trials, and use different genes to define the HRR deficiency (HRD) status. Thus, tumor and germline HRR PV rates may be subject to ascertainment bias.

The same heterogeneity is emerging in the molecular workflows chosen to identify HRR gene alterations, probably secondary to several emerging issues in the tumor testing.^24^ The DNA quality of tumor tissue results often compromised, as they come from small prostate biopsy, or old archival samples, distant from the contemporaneous molecular landscape.^25^^,^^26^ Genomic testing on metastatic tissue may better reflect the current molecular picture of the disease but is limited by high failure rates (16%-40%), especially for bone metastasis,^27–29^ which represents the predominant metastatic site in this tumor. In this scenario, plasma circulating tumor DNA (ctDNA) profiling of HRR genes, often referred to as liquid biopsy, has been recently implemented as a complementary diagnostic tool when tumor testing has failed, archival tissues are difficult to retrieve, or DNA quality of tumor sample is inadequate.^24^ Despite the ability to simultaneously capture both local and distant sites, and the reported high concordance between ctDNA and tumor tissue samples^30^^,^^31^ several limitations of ctDNA profiling, and gray zones on correct timing for blood collection, still exist.^32^

These important issues emphasize the clinical need to have a solid foundational understanding of genomic and testing data to support clinical practice recommendations on the optimal DNA source and timing of HRR tests.

The objective of this study was to investigate the somatic and germline HRR mutation prevalence, the type of testing among germline, tumor and/or ctDNA, the profiling of HRR or only *BRCA1/2*, and the timing of testing in the patient’s disease course, carried out as part of routine clinical care in a wide real-world Italian population of PC patients, since genetic testing and PARPis were incorporated into daily clinical practice.

## Methods

### Study design and population

This was a real-world, multicenter, observational, retrospective, study involving 20 Italian cancer centers. The study population included consecutive patients diagnosed with PC undergoing germline, tumor and/or ctDNA *BRCA1/2* or HRR testing between January 1 2020 and January 31, 2025. All included patients had a known genetic testing result showing the HRR or *BRCA1/2* mutational status. HRR mutation prevalence, only *BRCA1/2* testing vs HRR multigene panel, DNA source for testing (blood or saliva sample vs tumor tissue sample and/or ctDNA sample), type of tumor tissue for analysis (prostate biopsy vs radical prostatectomy vs metastatic tissue), site of metastatic tissue (lung vs bone vs liver vs lymph node vs other), and timing of testing (localized vs metastatic disease and hormone-sensitive vs castration-resistant metastatic disease), and *BRCA1/2* PV type (detected by tumor tissue vs ctDNA) were assessed. Multiple primary tumor (MPT) frequency and family cancer history were evaluated. The diagnostic testing pathway in the Italian health-care system is reported as Supplementary Material S1.

Clinical and pathological information were extracted, at each participating center, from the patients’ medical reports for clinical use.

The study protocol was approved on January 11th, 2024, by the Ethical Committee of the coordinating center (Comitato Etico Palermo 1 - University Hospital AOUP “Paolo Giaccone,” Palermo, – Italy—No. 01/2024, Study Protocol “PROGRESS”) and by the Institutional Review Boards of the participating centers.

### Statistical considerations

Descriptive analyses were used to assess patients’ characteristics. The prevalence of alterations in specific HRR genes was calculated as the proportion of patients with ≥1 positive testing result for a given gene alteration among PC patients subjected to genetic testing for that gene alteration. The differences between subgroups were evaluated by T-Student and chi-square tests. *P* values <.05 were considered statistically significant. Statistical analyses were conducted using IBM SPSS Statistic Software, Version 28.0 (IBM Corporation).

## Results

### Patients’ population

A total of 1400 PC patients were included in this real-world study. Median age was 66 (range 40-91) years. At PC diagnosis 496 cases (35.4%) showed organ-confined disease, while 168 (12.0%) presented locoregional metastasis, and 709 (50.6%) distant metastasis. A summary of the patient characteristics is reported in Table 1.

**Table 1.** Patient characteristics.

	Overall 1400 (100)
**Age, years (y)**	
**Median**	66
**Range**	40-91
**Stage at diagnosis**	
** Organ confined disease**	496 (35.4)
** Locoregional metastasis**	168 (12.1)
** Distant metastasis**	709 (50.6)
** Unknown**	27 (1.9)
**ISUP grade group**	
** ≤2**	125 (8.9)
** 3**	226 (16.1)
** ≥4**	972 (69.4)
** Unknown**	77 (5.5)
**Intraductal carcinoma (IDCP)**	282 (20.1)
**Radical prostatectomy for localized CSPC**	545 (38.9)
**Definitive radiotherapy**	231 (16.5)
**Metastatic stage**	
** Total**	1318
** De novo**	709 (53.8)
** Metachronous**	609 (46.2)
**Metastatic site**	
** Bone**	1068 (81.6)
** Visceral**	199 (15.2)
** Distant lymph nodes**	837 (63.9)
** Others**	57 (4.3)
**ECOG performance status**	
** 0**	928 (66.3)
** 1**	296 (21.1)

### The timing of testing

**Figure 1.**
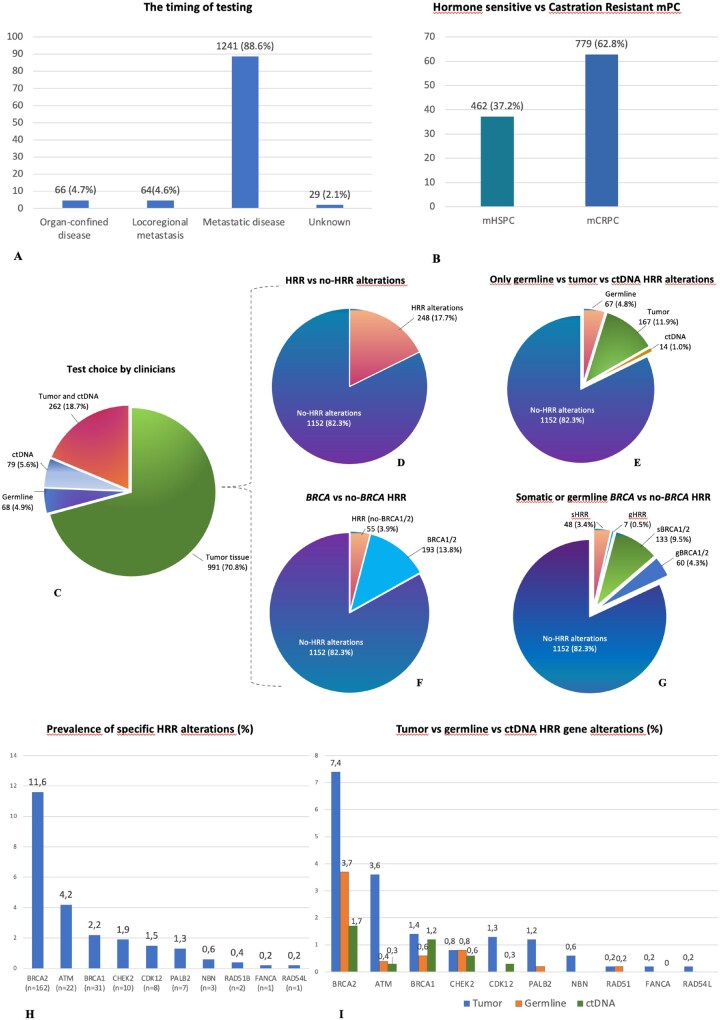
Timing of testing, test choice by clinicians and HRR genetic landscape in the study population. (A) Disease stage when the test was performed. (B) Number of tests according to the hormone-sensitive vs castration-resistant status of metastatic disease. (C) Test choice among tumor tissue and ctDNA, only tumor tissue, only ctDNA, and only germline, in the study population. Tumor and ctDNA testing positive were followed by germline testing, not included in the graphic. (D) HRR vs no-HRR alterations in the study populations. (E) Patients with only germline vs only tumor tissue vs only ctDNA HRR gene alterations. (F) *BRCA1/2* vs no-*BRCA1/2* HRR alterations. (G) Germline or somatic *BRCA1/2* alterations vs germline or somatic no-*BRCA1/2* HRR gene alterations. Somatic alterations included tumor and ctDNA alterations. (H) Prevalence of specific HRR alterations. (I) Tumor vs germline vs ctDNA alterations in the mHRR population.

In our study population, most patients had metastatic disease when tested. The number of patients tested with an organ-confined disease, locoregional metastatic disease, and distant metastatic disease was 66 (4.7%), 64 (4.6%), and 1241 (88.6%), respectively (Figure 1A). Patients tested with localized disease were patients with a cancer family history or early onset of PC and underwent germline *BRCA* testing. Patients tested after the diagnosis of metastatic disease were mainly subjected to somatic testing (tumor and/or plasma ctDNA testing) (1195, 96.3%).

When we assessed the prevalence of testing according to the hormone-sensitive vs castration-resistant state of metastatic disease, emerged that the majority of HRR testing was conducted during the mCRPC (779 patients, 62.8%) (Figure 1B).

### Somatic and/or germline test choice

Tumor tissue was undertaken first in 991 (70.8%) PC patients. In this group, only patients with PV identified via tumor testing were referred for germline testing and counseling. Tumor tissue testing and ctDNA testing were performed in 262 patients (18.7%), while a ctDNA testing without tumor testing was performed in 79 patients (5.6%). A germline testing only was provided in a subgroup of 68 patients (4.9%). Among these patients, 46 (67.6%) did not show preventive criteria, such as tumor history in family members, or early onset of tumors (≤65 years old); conversely, this subgroup of patients showed metastatic disease and indication to tumor and/or ctDNA testing. Thus, 3.3% of PC patients with tests performed for therapeutic purposes, missed the opportunity to detect somatic variants confined to the tumor (Figure 1C).

Only *BRCA1* and *BRCA2* genes were tested in 841 PC patients (61.8%); a multigene panel, including *BRCA1/2* and a variable number of other HRR genes, was performed in 520 patients (38.2%).

### Genetic landscape

A total of 248 (17.7%) PC patients showed germline or somatic alterations in the HRR genes (Figure 1D): 67 patients were carriers of germline PVs (4.8%), and 167 patients showed only tumor alterations (11.9%). Importantly, a subgroup of 14 patients showed alteration only in ctDNA (1%), while the tumor and/or germline testing were reported negative or inconclusive, with important therapeutic implications (Figure 1E).

Mutation prevalence in *BRCA1/2* genes was 13.8%, with 4.3% of germline PVs and 9.5% of somatic PVs (Figure 1F). In the mHRR population, the most frequent alterations were in the *BRCA2* gene (162, 11.6%), followed by *ATM* (22, 4.2%), *BRCA1* (31, 2.2%), *CHEK2* (10, 1.9%), *CDK12* (8, 1.5%), and *PALB2* (7, 1.3%). PVs in other genes were rare (Figure 1G).

Differences in the prevalence of germline vs tumor or ctDNA alterations were reported in the no-*BRCA1/2* HRR genes (Figure 1H**)**.

### Multiple primary tumors and tumor family history

Although in most PC patients, carriers of HRR gPV, only the prostate tumor diagnosis occurs, these individuals, as carriers of constitutional variants, may have an increased lifetime risk of developing multiple primary tumors over time.^33^ We reported the prevalence of previous tumor diagnosis, before the PC onset, in men carrying germline deleterious variants in *BRCA1/2,* or other susceptibility genes in the HRR pathway, and the tumor number and type in family members (Table 2 and Table S1).

**Table 2.** Median age, personal second tumor history, and tumor family history, in patients harboring germline PVs in *BRCA1/2* genes, in patients harboring germline PVs in HRR genes other than *BRCA1/2*, and in patients without any gPV in HRR genes.

	Overall *n*. (%)	*BRCA1/2* gPVs *n*. (%)	No-*BRCA1/2 HRR* gPVs *n*. (%)	HRR WT *n*. (%)	*P* value
**Number of PC patients**	1400 (100)	60 (4.3)	7 (0.5)	1333 (95.2)	-
**Median age at PC diagnosis**					
** Years (range)**	66 (40-91)	67 (47-87)	68 (54-84)	66 (40-91)	.8
**Personal second tumor history**					
** Yes**	128 (9.1)	8 (13.3)	1 (14.3)	119 (8.9)	.4
** No**	1272 (90.8)	52 (86.7)	6 (85.7)	1214 (91.1)	
**Family history of tumors**					
** ≥1 FDR**	520 (37.1)	31 (51.7)	5 (71.4)	484 (36.3)	.009
** ≥2 SDR**	76 (5.4)	8 (13.3)	2 (28.6)	66 (4.9)	<.001
** 1 SDR**	106 (7.6)	10 (16.7)	1 (14.3)	95 (7.1)	.01

Abbreviations: FDR, first-degree relative; HRR, homologous recombination repair; gPVs, germline (likely) pathogenic variants; SDR, second-degree relative; WT, wild type.

The frequency of MPT history in the overall PC population was 9.1% (128 patients). The most frequent second tumor site in the *BRCA1/2* gPV carriers was urothelial (3 patients, 37.5%), while colorectal was the most frequent site among the patients wild type (WT) for *BRCA1/2* or other HRR gPV (25 patients, 21%) (Table S1).

A total of 520 patients reported a family history of DNA-damage repair (DDR)-associated tumors in ≥1 first-degree relative (FDR) (37.1%), 76 patients in ≥2 in second-degree relative (SDR) (5.4%), and 106 patients in 1 SDR (7.6%). Family history of DDR tumors in at least 1 FDR of patients harboring gPVs in *BRCA1/2*, or HRR genes other than *BRCA1/2*, was significantly increased compared to the patients without any gPV in HRR genes (31 [51.7%] vs 5 [71.4%] vs 484 [36.3%], respectively; *P* = .009) (Table 2).

The DDR-associated tumor type in family members was reported in Table S1. Other tumors in family members (FDR and/or SDR), usually unrecognized as DDR-associated tumors, are reported in Table S2.

The median age at PC diagnosis within subgroups was reported in Table 2.

### Tumor tissue samples for genomic testing

**Figure 2.**
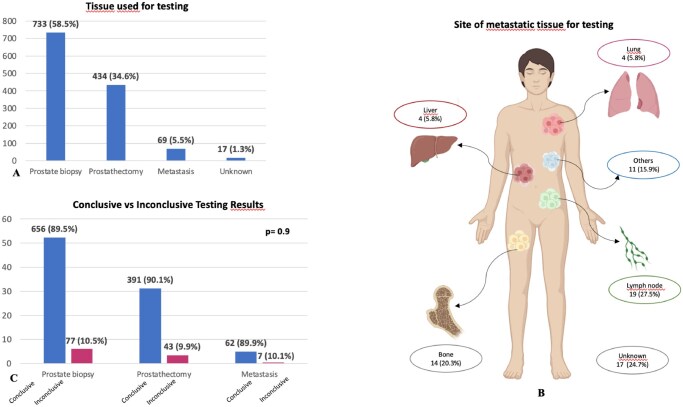
Tumor tissue samples chosen for genomic testing. (A) Type of tumor tissue used for genomic testing among prostate biopsy, prostatectomy, and metastasis. (B) Metastatic sites profiled for HRR testing. (C) Conclusive vs inconclusive testing results, according to the type of tumor sample.

Prostate biopsy was the most frequent sample used for the genomic testing (733 patients, 58.5%). Archival tumor tissue from prostate surgery, or metastatic samples, were used in 434 patients (34.6%), and 69 patients (5.5%), respectively (Figure 2A). Among metastatic sites, lymph nodes were the most prevalent (19 patients, 27.5%), followed by bone (14 patients, 20.3%) (Figure 2B).

The rate of conclusive results was 89.7% and varied widely according to the type of tumor tissue used for the analysis. The inconclusive result was reported for 77 patients (10.5%) of testing performed on prostate biopsy, 42 patients (9.9%) testing on tissue from prostatectomy, and 7 patients (10.1%) of testing on metastatic tumor tissue (Figure 2C). For the metastatic tumor tissue, inconclusive results were more frequent in the bone metastasis (5/14 tests, 35.7%) compared to other sites, particularly lymph node (1/19 tests, 5.3%) (Table S3).

### Metastatic hormone-sensitive versus castration-resistant PC

**Figure 3.**
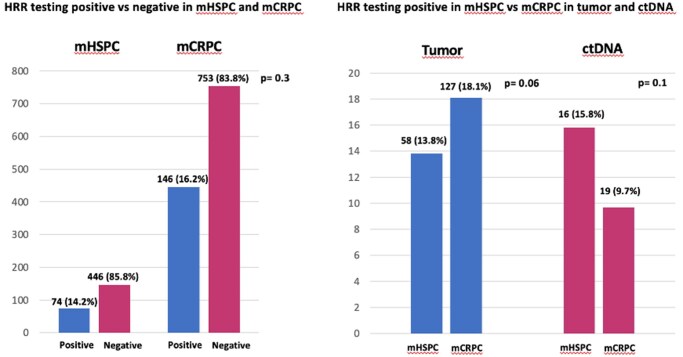
Testing positive in mCRPC and mHSPC (A) The prevalence of somatic alterations (testing positive vs negative) in the mCRPC compared to mHSPC. (B) The prevalence of somatic alterations (testing positive vs negative) in the mCRPC compared to mHSPC, detected on tumor and/or ctDNA.

The prevalence of somatic alterations, detected on tumor and/or ctDNA, was higher in the mCRPC, compared to mHSPC (146 [16.2%] vs 74 [14.2%], respectively), despite this difference was not statistically significant (*P* = .3) (Figure 3A). This finding was confirmed when we evaluated only the patients who underwent tumor tests, where the mCRPC patients showed a higher prevalence of HRR testing positive than mHSPC (127 [18.1%] vs 58 [13.8%], respectively; *P* = .06]) (Figure 3B). Conversely, when we evaluated the ctDNA testing, the prevalence of HRR alterations was lower in the mCRPC than in mHSPC patients (19 [9.7%] vs 16 [15.8%], respectively; *P* = .1) (Figure 3B).

### Circulating tumor DNA

**Figure 4.**
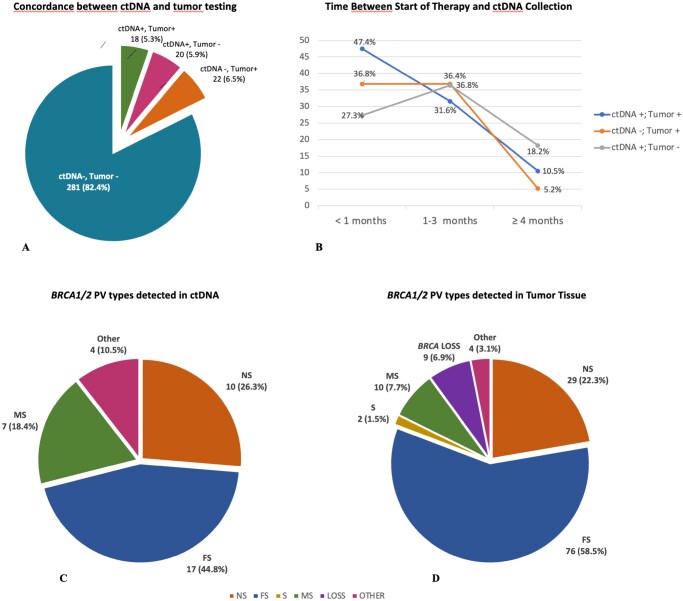
ctDNA testing. (A) Concordance between ctDNA and tumor tissue testing. (B) The time between the start of the latest systemic therapy of the patients and the plasma ctDNA collection. (C) *BRCA1/2* PV types detected on ctDNA. (D) *BRCA1/2* PV types detected on tumor tissue.

Patients tested with plasma ctDNA were 341 (24.3%). Patients tested with ctDNA and tumor testing were 262 (76.8%). The concordance between tumor testing and ctDNA was 83.9%. Patients with ctDNA positive and tumor testing positive were 18 (6.9%); patients with ctDNA negative and tumor testing negative were 202 (77.1%); patients with ctDNA negative and tumor testing positive were 30 (11.4%). Interestingly, 12 PC patients (4.6%) reported the ctDNA testing positive but tumor testing negative, leading to important therapeutic implications (Figure 4A).

To assess the optimal timing for ctDNA testing, we evaluated the time between the start of the latest systemic therapy of the patients and the plasma ctDNA collection. We categorized the patients into 3 groups: PC patients with <1 month, 1-3 months, or ≥ 4 months from the start of a new line of systemic treatment and the blood collection for ctDNA analysis.

Notably, a higher number of ctDNA testing positive, with the identification of HRR alterations, was obtained when the specimens were collected within 1 month before the initiation of a new line of therapy (Figure 4B). Overall, the prevalence of positive testing leading to the identification of mHRR, was 47.4%, 31.6%, and 10.5%, for testing within 1 month, between 1-3 months, and over 3 months, respectively, in the group of ctDNA positive and HRR positive, and was 27.3%, 36.8%, and 18.2%, in the group of patients with ctDNA positive and tumor negative (Figure 4B).

### *BRCA1/2* PV type and function

We also analyzed the *BRCA1/2* PV types detected on ctDNA vs tumor testing. PVs were grouped as frameshift (FS), nonsense (NS), missense (MS), splice site (SP), *BRCA* copy loss (Loss), and other. In the group of 341 patients tested on ctDNA, 30 (8.8%) showed *BRCA1/2* PVs. The PV type was available for 25 patients: 7 NS (28.0%), 11 FS (44.0%), 2 SP (8.0%), 4 MS (16.0%), 0 Loss (0.0%), and 1 other (premature truncating codon) (4.0%) (Figure 4C). In the group of 1253 patients tested on tumor tissue, 160 (12.8%) showed *BRCA1/2* PVs. The PV type was available for 130 patients: 29 NS (22.3%), 76 FS (58.5%), 2 SP (1.5%), 10 MS (7.7%), 9 Loss (6.9%), and 4 other PVs (3.1%) (Figure 4D).

## Discussion

The genomic and treatment landscape of PC has become increasingly complex. *BRCA1/2* somatic or germline alterations confer therapeutic sensitivity to PARPis, highlighting the current critical role that profiling the *BRCA1/2*, and the other genes in the HRR pathway, has in the optimal treatment selection and management of PC patients.^34^^,^^35^

Initially, the workflow developed in molecular testing approaches for identifying HRR gene alterations was adapted from breast and ovarian cancer testing pathways.^1^ Recently, peculiar issues, specific to PC, have constantly emerged, presenting several challenges for clinicians. Particularly, the optimal timing of testing, and the optimal DNA source for *BRCA* gene profiling, are the focus of ongoing debates to increase the success rate of *BRCA* analysis.^36–38^

Our data derives from a real-world study on 1400 PC patients who underwent germline, tumor, and/or ctDNA testing for the evaluation of *BRCA1/2* or the full HRR genes mutational status, with the aim to provide information on the genetic landscape, and the associated diagnostic testing pathways, in a large population of PC patients followed in 20 centers representative of the Italian health public system.

In our study population, tumor tissue was undertaken first in 991 patients (70.8%). Germline testing, in the absence of preventive criteria, was undertaken alone in 46 patients (3.3%) lacking, in this subgroup, the therapeutic opportunity to treat patients with PVs confined to the tumor. According to the main international guidelines, germline testing, that is capable of identifying only inherited PVs, should be proposed for patients affected by metastatic or localized PC having a high risk of carrying germline *BRCA* PVs based on personal or family cancer history.^24^^,^^26^^,^^39^ The percentage of 3.3% of tests performed only as germline in the therapeutic setting, although low, reflects the need to further implement and ameliorate the tumor testing paths in clinical practice.

Currently, tumor testing represents the gold standard for identifying HRR gene variants in the therapeutic setting, to inform clinical decisions regarding eligibility for PARPis.^24^ Because *BRCA* tumor testing is not able to discriminate tumors from constitutional PVs, any *BRCA* PV found in tumor tissue must be confirmed through germline testing. Our real-world data showed that confirmatory germline testing in the case of positive tumor testing was not an established practice. Thus, the prevalence of germline alterations could be underestimated. It is important to point out that, although tumor testing is the most practical approach at present, the tumor-only analysis presents also limitations, missing 8%-17% of germline *BRCA* PVs for technical issues.^40^^,^^41^ Indeed, recent evidences showed that germline PVs in PC can have low variant allele frequency on tumor sequencing, under the thresholds for follow-up germline testing.^42^ As a consequence, based on the current technologies, concurrent tumor and germline testing could ensure the comprehensive detection of the relevant HRR alterations, as well as recommended from same guidelines.^1^^,^^24^

Another important clinical issue is the literature evidence of a high proportion of uninformative testing results when the *BRCA*/HRR testing is performed on tumor tissue. Data from PROfound study reported 58% of conclusive results from tumor tissue,^22^ even though further studies showed higher numbers of tumor samples successfully sequenced.^43–45^ In the current, real-world, study population, the rate of conclusive results was 89.7%. Inconclusive results were reported in the 10.5%, 9.9%, and 10.1% of testing performed on prostate biopsy, prostatectomy, and metastatic tumor tissue, respectively. In the subgroup of testing performed on the metastatic tumor tissue, inconclusive results were particularly frequent in bone metastasis, reaching 35.7%. While in the lymph node tissue, the number of inconclusive tests was significantly lower (5.3%). These findings confirmed the success rate reported in the PROfound study, where genomic testing on bone metastases had the lowest rate of conclusive results (42.6%) than lymph node samples (74.7%).^27^

In this context, it is still unclear the optimal time point during the patient’s disease course at which to conduct the somatic test. Particularly, it remains debated if the genomic testing should be performed at diagnosis of metastatic disease, as HRR gene alterations are generally acquired early truncal events, or at the onset of the mCRPC, according to the scientific rationale that cancer genome evolve over time, shaped by several years of treatment.^46^ Emerging data showed a high concordance for genomic aberrations between primary and castration-resistant matched tumor tissue, supporting the hypothesis that HRR alterations are early truncal events, developed even before exposure to androgen deprivation.^47^^,^^48^ According to this evidence, our data did not show an important difference in somatic alterations between mCRPC and mHSPC: these data may become of interest due to the possible landing of treatment combination in the mCSPC setting. Among the tumor tests, the prevalence of HRR alterations was higher in the mCRPC population (mCRPC vs mHSPC, 18.1% vs 13.8%, respectively; *P* = .06), although the difference did not reach statistical significance. This finding could be partly attributed to the poorer prognosis associated with the *BRCA* alterations, which leads to a higher likelihood of progression to castration-resistant disease.

According to this observation, performing *BRCA* testing on primary tumor samples, collected at PC diagnosis, may be appropriate after disease progression to mCRPC if the storage is under optimal conditions; while, it is preferable to avoid performing tumor testing on old archival tissue, considering re-biopsy of metastatic sites.

In this scenario, ctDNA could be the ideal biomarker for providing a comprehensive and dynamic “snapshot” of the tumor, overcoming the limitations of tumor tissue testing. Our daily clinical practice results showed a concordance rate between tumor and ctDNA testing of 83.9%. Data from the PROfound clinical trial showed 81% of positivity agreement for *BRCA1*, *BRCA2*, and *ATM* alterations between tumor tissue and ctDNA.^49^ A higher agreement was in the largest study of ctDNA in PC. In this study were profiled tissue and plasma from more than 3.000 mCRPC patients, including 1.674 from TRITON 2/3 clinical trials.^30^ The results reported that 94% of *BRCA1/2* PVs and 90% of all *BRCA1/2* variants detected by tumor tissue testing were also found in ctDNA.^30^

Notably, in the current study population, a higher number of ctDNA testing positive, with the identification of HRR alterations, was obtained when the specimens were collected within 1 month before the initiation of a new line of therapy. In these patients the prevalence of positive testing was 47.5% when the time between ctDNA collection and the start of a new line of therapy was < 1 month, and 10.5% when this interval was > 3 months. This observation is consistent with the hypothesis that progressing tumors will shed more DNA into circulation and that effective treatments can rapidly reduce the ctDNA fraction, potentially leading to inconclusive testing results.^32^ These results are consistent with recent literature data showing that ctDNA fraction correlates with tumor burden and is highest in patients with liver metastases, and more advanced lines of treatment.^50^ This relevant finding supports the recommendations to prioritize ctDNA testing at times of progression.

One main finding with potential clinical implications was the relatively high number of PC patients (4.6%) reporting the tumor testing negative but ctDNA testing positive. This subgroup of patients, only through liquid biopsy testing, had access to PARPi treatment. There are several potential explanations of this finding. First, the aforementioned technical issues of tumor testing, which can lead to false negatives. Second, the mutational profile may change on tumor progression. Accordingly, a recent study showed that 11% of mCRPC patients harbored new somatic gene alterations on repeat NGS testing, predominantly ctDNA.^51^

When we compared the *BRCA1/2* PV types detected on ctDNA or tumor testing, the *BRCA2* copy loss were detected only by tumor tissue testing. This observation highlights the significance of *BRCA* alteration type in determining the sensitivity and detection of specific genetic alterations in liquid biopsy.

Difficult to contextualize is the prevalence of somatic alterations detected on ctDNA in the mCRPC compared to mHSPC. Despite the difference between the 2 groups was not statistically significant, the HRR alterations were lower in the mCRPC than in mHSPC patients (9.7% vs 15.8%, respectively; *P* = .1). The scientific background of this result remains speculative, with few literature data on HRR alteration on ctDNA in hormone-sensitive vs castration-resistant disease. The dynamics of ctDNA release mechanisms are still not fully understood; ctDNA levels are affected by dynamic changes during therapy and vary according to treatment response or progression.^51^ Furthermore, many clinical and technical variables may preclude the detection of important clinically relevant alterations such as somatic *BRCA2* truncating mutations and homozygous deletions,^52^^,^^53^^,^^54^ conditioning the number of gene alterations detected.

The prevalence of *BRCA1/2* germline PVs in the current, wide, Italian study population was 4.3%. According to the literature data, the prevalence of *BRCA1/2* somatic alteration was higher than germline (9.5%).^55^ Overall, 17.7% of patients showed somatic or germline alterations in the HRR pathway genes.

In the context of constitutional variants, a finding of clinical interest was the relatively high number of DDR-associated tumor types in family members in the group of patients wild-type for any gHRR PVs. Among these PC patients, 36.3% showed at least 1 FDR with tumor history in the spectrum of Lynch Syndrome. These findings suggest the possible involvement of non-HRR, unrecognized genes, in the onset of the cancers in these families, and underline the need to expand the clinical practice of the number of genes to profile in PC tumors. In fact, in this study population, a multigene panel including HRR other than *BRCA1/2* was performed only in 520 patients (38.2%).

This study has several strengths, including the large sample size. Importantly, to our knowledge, it is the largest real-world, cohort study, in PC patients who underwent *BRCA*/HRR genomic testing. There are very few studies evaluating the workflow adopted in clinical practice, and even today, data on *BRCA*/HRR testing come primarily from clinical trials. Data in the current study expand the understanding of several issues associated with *BRCA*/HRR genomic testing in prostate cancer patients and may represent the scientific, real-world, background, for optimal strategies to minimize false positives or negatives in testing results. Furthermore, this is the first study that allows us to know the prevalence of germline mutations in a wide Italian study population.

We also acknowledge some limitations of the study. Mainly, the retrospective data analysis. However, even though retrospective, the analysis of PC consecutive patients, allowed us to have a reliable estimate of *BRCA* and HRR gene prevalence, and a broad picture of *BRCA*/HRR testing paths chosen by clinicians in the Italian health system.

## Conclusions

Currently, test choice for *BRCA*/HRR profiling is mainly based on the clinical context, availability of the types of testing, and suitability of tumor tissue, all variables influencing the success rate of genomic testing. Optimize *BRCA*/HRR testing, choosing the optimal DNA source, disease state, and timing for tissue and blood collection, can allow to draw and monitor virtuous workflows for molecular testing approaches to maximize the detection and understanding of clinically relevant HRR gene alterations.

## Supplementary Material

oyaf395_Supplementary_Data

## Data Availability

The data underlying this article are available in the article and in its online supplementary material.
