# Supplementary material for: Homologous recombination repair genetic testing variables and diagnostic paths for prostate cancer patients: a multicenter cohort study
Source: Oncologist. 2025 Dec 2;30(12):oyaf395. doi: 10.1093/oncolo/oyaf395 (PMC12921449; doi:10.1093/oncolo/oyaf395)
Supplement: oyaf395_Supplementary_Data [file oyaf395_supplementary_data.docx]

**Supplementary Tables**

**Supplementary Table 1.** Tumor type in patients showing personal second tumor history and tumor family history, according to the presence of germline PVs in BRCA1/2 genes, HRR genes other than BRCA1/2, or the absence of any germline PV in HRR genes.

|  | **Overall**  **n. (%)** | ***BRCA1/2* gPVs n. (%)** | **No-*BRCA1/2 HRR* gPVs**  **n. (%)** | **HRR WT**  **n. (%)** |
| --- | --- | --- | --- | --- |
| **Second tumor site**   - Colorectal cancer - Urothelial cancer - Melanoma - Kidney cancer - Lung cancer - Lymphoma - Skin cancer (no melanoma) - Gastric cancer - Breast cancer - Soft Tissue Sarcoma - Others | 26 (20.3)  21 (16.4)  13 (10.2)  12 (9.4)  9 (7)  7 (5.5)  6 (4.7)  4 (3.1)  4 (3.1)  4 (3.1)  31 (24.2) | 1 (12.5)  3 (37.5)  1 (12.5)  \  \  \  \  \  1 (12.5)  \  2 (25) | \  \  \  \  \  \  \  \  \  \  1 (100) | 25 (21)  18 (15.1)  12 (10.1)  12 (10.1)  9 (7.6)  7 (5.9)  6 (5)  4 (3.4)  3 (2.5)  4 (3.4)  28 (23.5) |
| **DDR-associated tumor type in family members**   - Prostate cancer - Breast cancer - Colorectal cancer - Gastric cancer - Pancreatic cancer - Urothelial cancer - Endometrial cancer - Ovarian cancer - Melanoma | 242 (17.3)  150 (10.7)  61 (4.4)  54 (3.9)  36 (2.6)  28 (2)  25 (1.8)  25 (1.8)  9 (0.6) | 14 (23.3)  18 (30)  2 (3.3)  5 (8.3)  7 (11.6)  1 (1.7)  \  9 (15)  1 (1.7) | 4 (57.1)  2 (28.6)  \  1 (14.3)  1 (14.3)  \  1 (14.3)  \  \ | 224 (16.8)  130 (9.8)  59 (4.4)  48 (3.6)  28 (2.1)  27 (2)  24 (1.8)  16 (1.2)  8 (0.6) |
| **Number of family members with DDR-associated tumor type**   - Prostate cancer - Breast cancer - Colorectal cancer - Gastric cancer - Pancreatic cancer - Ovarian cancer - Endometrial cancer - Urothelial cancer - Melanoma | 318 (22.7)  210 (15)  64 (4.6)  58 (4.1)  43 (3.1)  31 (2.2)  28 (2)  28 (2)  10 (0.7) | 18 (30.6)  32 (51.6)  2 (3.2)  5 (8.1)  10 (16.1)  14 (22.6)  \  1 (1.6)  1 (1.6) | 6 (85.7)  5 (71.4)  \  1 (14.3)  1 (14.3)  \  1 (14.3)  \  \ | 294 (22.1)  173 (13)  62 (4.7)  52 (3.9)  32 (2.4)  17 (1.3)  27 (2)  27 (2)  9 (0.7) |

DDR, DNA Damage Repair; HRR, Homologous Recombination Repair; gPVs, germline (Likely) Pathogenic Variants; WT, Wild Type.

**Supplementary Table 2**

|  | **Overall**  **1400 (100)** | ***BRCA1/2* gPVs 60 (4.3)** | **No-*BRCA1/2 HRR* gPVs**  **7 (0.5)** | **HRR WT**  **1333 (95.2)** |
| --- | --- | --- | --- | --- |
| **Site of other tumors in family members (FDR and/or SDR)**   - Lung - Liver - Head-neck - Leukemia - Brain - Kidney - Lymphoma - Intestine - Thyroid - Esophagus - Multiple myeloma - Soft Tissue - Biliary tract - Penis - Parotid - Ovarian bordeline - Papilla of Vater - Parathyroid - Peritoneum - Pleura - Testes | 94 (6.7)  24 (1.7)  19 (1.4)  14 (1)  9 (0.6)  9 (0.6)  8 (0.6)  7 (0.5)  6 (0.4)  5 (0.4)  5 (0.4)  4 (0.3)  3 (0.2)  3 (0.2)  2 (0.1)  1 (0.1)  1 (0.1)  1 (0.1)  1 (0.1)  1 (0.1)  1 (0.1) | 4 (6.7)  2 (3.3)  \  \  \  2 (3.3)  \  \  2 (3.3)  \  \  1 (1.7)  \  \  \  \  \  1 (1.7)  1 (1.7)  \  \ | 2 (28.6)  1 (14.3)  \  \  1 (14.3)  \  \  \  \  \  1 (14.3)  1 (14.3)  \  \  \  \  \  \  \  \ | 88 (6.6)  21 (1.6)  19 (1.4)  14 (1)  8 (0.6)  7 (0.5)  8 (0.6)  7 (0.5)  4 (0.3)  5 (0.4)  5 (0.4)  2 (0.1)  2 (0.1)  3 (0.2)  2 (0.1)  1 (0.1)  1 (0.1)  \  \  1 (0.1)  1 (0.1) |
| **Number of family members with other tumors (FDR and/or SDR)**   - Lung - Liver - Head-neck - Leukemia - Brain - Kidney - Lymphoma - Intestine - Thyroid - Esophagus - Multiple myeloma - Soft Tissue - Biliary tract - Penis - Parotid - Ovarian bordeline - Papilla Vater - Parathyroid - Peritoneum - Pleura - Testes | 104 (7.4)  28 (2)  19 (1.4)  14 (1)  10 (0.7)  9 (0.6)  8 (0.6)  7 (0.5)  6 (0.4)  5 (0.4)  5 (0.4)  4 (0.3)  3 (0.2)  3 (0.2)  2 (0.1)  1 (0.1)  1 (0.1)  1 (0.1)  1 (0.1)  1 (0.1)  1 (0.1) | 5 (8.3)  2 (3.3)  \  \  \  2 (3.3)  \  \  2 (3.3)  \  \  1 (1.7)  \  \  \  \  \  1 (1.7)  1 (1.7)  \  \ | 2 (28.6)  1 (14.3)  \  \  1 (14.3)  \  \  \  \  \  \  1 (14.3)  1 (14.3)  \  \  \  \  \  \  \  \ | 92 (6.9)  25 (1.9)  16 (1.2)  13 (1)  9 (0.7)  7 (0.5)  8 (0.6)  7 (0.5)  4 (0.3)  5 (0.4)  5 (0.4)  2 (0.1)  2 (0.1)  3 (0.2)  2 (0.1)  1 (0.1)  1 (0.1)  \  \  1 (0.1)  1 (0.1) |

FDR; First-Degree relative; SDR, Second-Degree Relative; WT, Wild Type

**Supplementary Table 3**

| **Site of metastatic tissue for testing** | **Number of Testing**  **n. (%)** | **Number of inconclusive results** |
| --- | --- | --- |
| Bone | 14 (20.3) | 5 (35.7) |
| Lung | 4 (5.8) | 0 (0) |
| Liver | 4 (5.8) | 1 (25) |
| Lymph node | 19 (27.5) | 1 (5.3) |
| Others | 11 (15.9) | 1 (9.1) |
| Unknown | 17 (24.7) | 0 (0) |

**Supplementary Material 1**

In Italy, genetic testing is currently recommended for 2 populations: (1) Germline testing with preventive purpose for patients affected by metastatic or non-metastatic PC with familial risk; (2) Somatic Testing with therapeutic purpose for patients affected by metastatic PC. In the therapeutic setting, pre-test consultation is carried out by healthcare providers (medical oncologists, urologists, and radiation oncologists), who provide detailed information on the clinical and therapeutic implications of potential testing outcomes, and the genetic implications of identifying a somatic BRCA1/2 likely pathogenetic variant/pathogenetic variant (LPV/PV). If somatic BRCA1/2 LPV/PV is identified, germline testing is offered; when germline BRCA1/2 LPV/PV is detected, or familial risk criteria are met, patients are referred for genetic counseling and post-test consultation (1, 26).
